# Lens Aquaporins in Health and Disease: Location is Everything!

**DOI:** 10.3389/fphys.2022.882550

**Published:** 2022-04-19

**Authors:** Kevin L. Schey, Romell B. Gletten, Carla V. T. O’Neale, Zhen Wang, Rosica S. Petrova, Paul J. Donaldson

**Affiliations:** ^1^ Department of Biochemistry, School of Medicine, Vanderbilt University, Nashville, TN, United States; ^2^ Department of Physiology, School of Medical Sciences, University of Auckland, Auckland, New Zealand

**Keywords:** cataract, protein aging, aquaporin regulation, microcirculation system, water transport

## Abstract

Cataract and presbyopia are the leading cause of vision loss and impaired vision, respectively, worldwide. Changes in lens biochemistry and physiology with age are responsible for vision impairment, yet the specific molecular changes that underpin such changes are not entirely understood. In order to preserve transparency over decades of life, the lens establishes and maintains a microcirculation system (MCS) that, through spatially localized ion pumps, induces circulation of water and nutrients into (influx) and metabolites out of (outflow and efflux) the lens. Aquaporins (AQPs) are predicted to play important roles in the establishment and maintenance of local and global water flow throughout the lens. This review discusses the structure and function of lens AQPs and, importantly, their spatial localization that is likely key to proper water flow through the MCS. Moreover, age-related changes are detailed and their predicted effects on the MCS are discussed leading to an updated MCS model. Lastly, the potential therapeutic targeting of AQPs for prevention or treatment of cataract and presbyopia is discussed.

## Introduction

The majority of cases of age-related vision loss in the world today are associated with the lens pathologies presbyopia and cataract (Frick et al., 2015). Presbyopia is the loss of the ability of the lens to dynamically change its shape (accommodate) to focus on near objects (Glasser and Kaufman, 1999), while cataract is the loss of the transparent properties of the lens (Asbell et al., 2005). Although it is believed these two lens pathologies are linked by oxidative damage to lens proteins (Kupfer, 1985), antioxidant-based therapies have to date proven ineffective in slowing the onset of either presbyopia or cataract (Braakhuis et al., 2019). Confirmation of the existence of a lens microcirculation system (MCS) (Vaghefi et al., 2011; Candia et al., 2012; Vaghefi and Donaldson, 2018), which generates a circulating flux of water, provides an alternative view not only to understand the onset of presbyopia and cataract, but also opens new therapeutic pathways to treat these lens pathologies.

It has been shown in animal lenses that movement of water through the lens generates a substantial and highly regulated pressure gradient (Gao et al., 2015), delivers nutrients and antioxidants to the lens center (Vaghefi and Donaldson, 2018), controls lens water content and volume (Donaldson et al., 2009), and maintains overall lens optics (Vaghefi et al., 2015). It is envisaged that in the human lens, these processes become dysfunctional with advancing age, and manifest first during middle age as presbyopia and then as cataract in the elderly. Consistent with this view, it has been shown that the free water content of the human lens increases with age (Lie et al., 2021) and is significantly increased in cataratous lenses (Bettelheim et al., 1986; Heys et al., 2008). Motivated by this growing recognition of the importance of water transport in the maintenance of the transparency and refractive properties of the lens, in this review we have focused on the role of water channels from the aquaporin (AQP) protein family in mediating, directing and regulating water flow in different regions of the lens. Since AQPs mediate the passive diffusion of water across cell membranes, the direction and magnitude of which is determined by osmotic gradients established across the membrane (Knepper, 1994; Verkman et al., 1996), we will first provide a review of the microcirculation system that generates the ion fluxes that drive water transport in the lens, before concentrating on how the expression of different lens AQPs and regional differences in their subcellular distribution, post-translational modification and regulation all contribute to lens water transport. We hypothesize that these regional differences, specifically the tissue and cellular locations of AQPs and their modifications facilitate water movement throughout the lens *via* the MCS. Further, we hypothesize that age-related changes in AQP structure and function lead to cataract formation. Finally, we will discuss therapeutic strategies being developed in other tissues that are targeting AQPs with the view to inform efforts to develop novel therapies on the lens.

## Lens Water Transport

**FIGURE 1:**
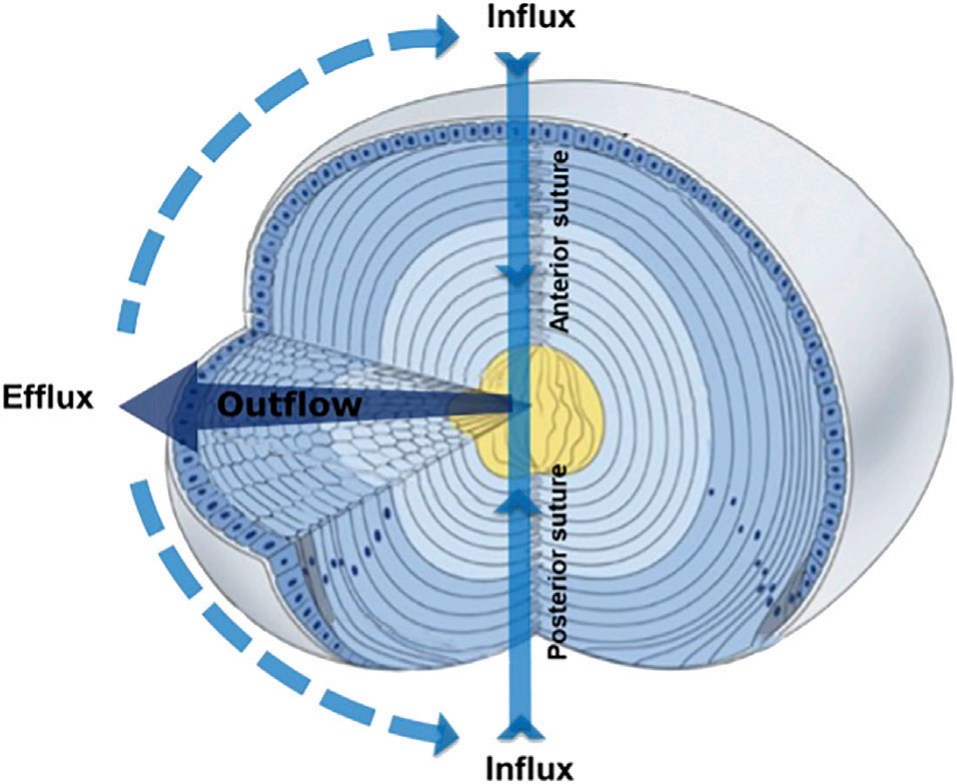
3D representation of the microcirculation model showing ion and fluid fluxes that enter the lens at both poles via the extracellular space (Influx), before crossing fiber cell membranes and exiting via an intercellular outflow pathway mediated by gap junctions, that directs the fluxes to the equatorial efflux zone where they exit the lens. Adapted from Shi et al., 2009.

To compensate for the lack of a blood supply, the lens operates an internal MCS (Figure 1) that delivers nutrients to deeper fiber cells, maintains fiber cell ionic homeostasis, and actively preserves the transparent and optical properties of the lens (Donaldson et al., 2017; Mathias et al., 2007; Mathias et al., 1997). The MCS is generated by a circulating current of Na^+^ ions, which primarily enters the lens at the poles and travels into the lens via the extracellular spaces between fiber cells (Figure 1, Influx). Na^+^ ions cross fiber cell membranes and return towards the surface *via* an intercellular outflow pathway mediated by gap junction (GJ) channels (Cx46 & Cx50) (Figure 1, Outflow), where Na^+^ is actively removed by Na^+^ pumps concentrated at the lens equator (Figure 1, Efflux). This circulating ion current generates a near isotonic water flow that enters the lens at both poles and exits at the equator (Candia et al., 2012). This flow of water has two consequences. First, the extracellular flow of water convects nutrients and antioxidants towards the deeper lying fiber cells where multiple membrane transporters enable cellular uptake (Donaldson and Lim, 2008). Mapping this extracellular delivery of solutes to the lens cores using MRI (Vaghefi et al., 2012) and confocal microscopy (Grey et al., 2003; Lim et al., 2009) has revealed that a barrier to extracellular diffusion forms in the inner cortex of the lens where mature fiber cells have lost their organelles. Through the use of spatially-resolved quantitative proteomics it has been shown that this closing of the extracellular space is accompanied with increased expression of gap junction, adherens junction and tight junction related proteins and by increases in the expression of AQP0 and its interaction partners, ezrin-radixin-moesin (ERM) proteins (Wang et al., 2021). It has been proposed that this barrier, by restricting radial extracellular space diffusion in the inner cortex, serves to preferentially direct water and solute fluxes into the lens nucleus *via* the sutures (Vaghefi et al., 2012).

**FIGURE 2:**
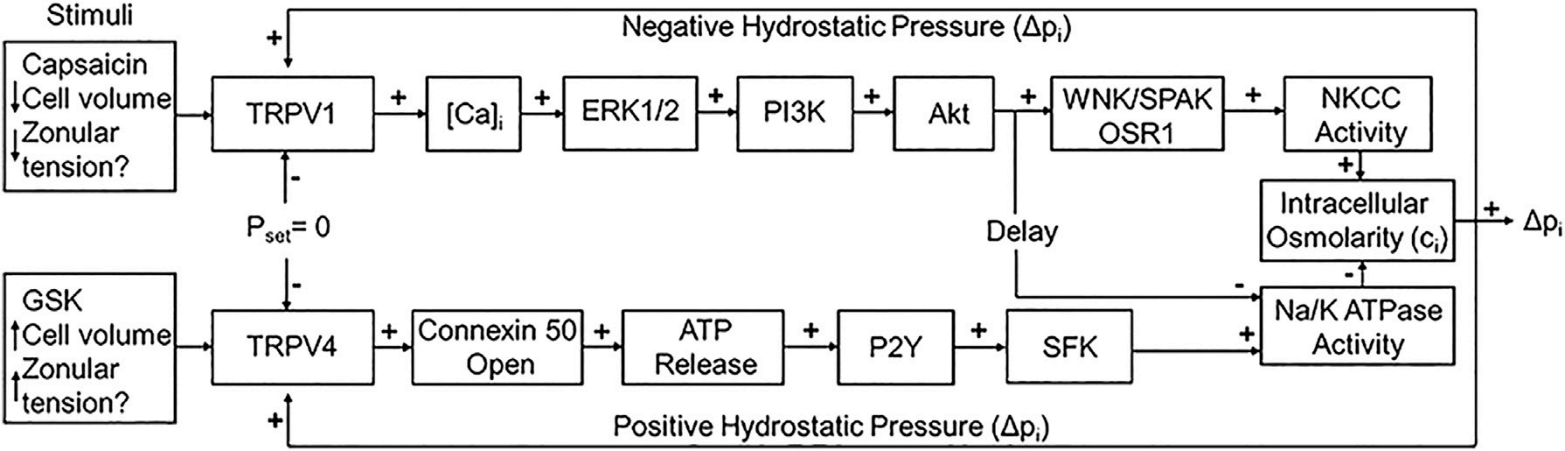
The dual-feedback control system that maintains hydrostatic pressure in the lens. Lens surface pressure (pset) is maintained by the competing activities of the two arms of a dual-feedback system that regulate ion transporters that control the intracellular osmolarity of cells at the lens surface. Increases in pressure (Dp_i_), hypoosmotic stress, increased zonular tension, or the TRPV4 agonist GSK1016790A (GSK), all work *via* TRPV4 to activate a signaling pathway that involves the release of ATP via hemichannel, the subsequent activation of purinergic P2Y receptors, and the Src family of protein tyrosine kinases (SFK) to increase the activity of the Na+/K- ATPase and decrease lens pressure. Decreases in pressure (Dp_i_), hyperosmotic stress, decreased zonular tension or the TRPV1 agonist capsaicin all work via TRPV1 to activate the extracellular signal-regulated kinase 1/2 (ERK1/2), phosphatidylinositol 3-kinase (PI3K/Akt) and the WNK (Kinase with no lysine (K)), and SPAK (Ste20-related proline-alanine-rich kinase)/OSR1(oxidative stress-responsive kinase-1) signaling pathway to directly activate the sodium potassium dichloride cotransporter (NKCC) and to eventually reduce the decrease in the activity of the Na+/K- ATPase to effect an increase in surface pressure. This scheme is based on earlier model (Gao et al., 2015; Shahidullah et al., 2018). The figure and figure caption are reused with no special permission under an open access Creative Common CC BY license published by MDPI from (Nakazawa et al., 2021).

Secondly, Mathias et al. have shown that the removal of water from the centre of the lens across the extracellular diffusion barrier, through an intercellular outflow pathway thought to be mediated by gap junction channels, generates a substantial hydrostatic pressure gradient, which ranges from 0 mmHg in the periphery to 335 mmHg in the lens centre in all lenses studied to date (Gao et al., 2013; Gao et al., 2011). This lens pressure gradient is maintained through a dual feedback system that utilizes the mechanosensitive Transient Receptor Potential Vanilloid channels, TRPV1 and TRPV4, to sense changes in pressure at the surface of the lens (Gao et al., 2015). TRPV1 and TRPV4 channels sense decreases and increases, respectively, in lens pressure and utilize distinct signalling pathways to modulate Na^+^ pump (Gao et al., 2015) and NKCC1 (Shahidullah et al., 2018) activity to ensure that a constant hydrostatic pressure and therefore water content is maintained (Figure 2). Altering the tension applied to the mouse lens via the zonules, through pharmacological modulation of ciliary muscle contractility, alters lens surface pressure and this change in pressure is mediated by TRPV1 and TRPV4 channels (Chen et al., 2019). This finding suggests that the hydrostatic pressure gradient, and therefore steady state water transport, in the non-accommodating mouse lens can be altered by contraction of the ciliary muscle.

In this scheme, the active transport of Na^+^ drives a directed isotonic flow of fluid through the lens. Near isotonic fluid flow, in turn, requires a high membrane permeability to water that is known to be mediated by the aquaporin family of water channels (Knepper, 1994; Verkman et al., 1996). However, what is not known is how different lens AQPs, which exhibit distinctly different functional properties, regulation mechanisms, and expression patterns, contribute to the generation of the microcirculation system. To address this knowledge gap we first discuss general properties and functions of AQPs before comparing the expression patterns of three lens AQPs and their modified forms. Finally, we detail lens AQP functional properties and how these properties are regulated in order to develop a working model on how the different AQP isoforms contribute to the generation of the lens microcirculation system which is so central to the maintenance of lens homeostasis.

## Aquaporin Structure and Function

**FIGURE 3:**
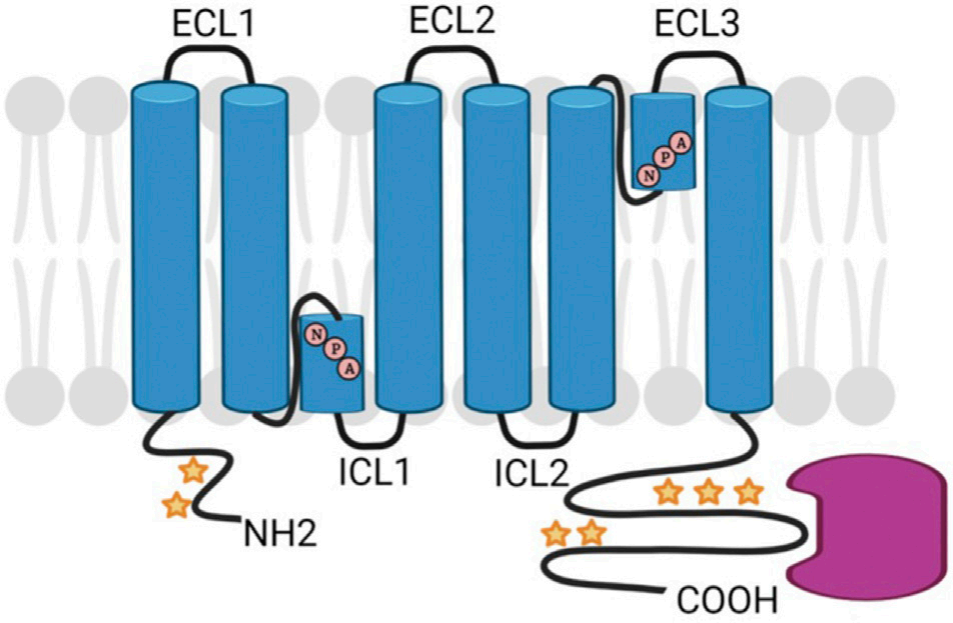
Schematic diagram of generic AQP structure showing NPA sequence motifs and regions of post-translational modification (stars) and protein interactions (pink). Created with BioRender.com.

Aquaporins are a class of transmembrane proteins that function as water channels. These water channels exist in 13 known isoforms (AQP0-AQP12) in mammals and act to move water bidirectionally across biological membranes through osmotically driven passive diffusion (Ishibashi et al., 2011; Rojek et al., 2008). Clearly, water transport is an essential cellular function as evidenced by the wide and diverse tissue-specific expression of aquaporins. For example, AQPs show wide-ranging distribution throughout tissues such as the eye, brain, kidney, liver, and heart with varying physiological functions in each tissue (Azad et al., 2021). Aquaporin isoforms display many structural similarities including six transmembrane domains and two conserved asparagine-proline-alanine (NPA) motifs that are associated with water transport (Maurel et al., 2015) (Figure 3). However, there is a subfamily of aquaporins, coined the superaquaporins, that show sequence deviation from the greater family, particularly in the NPA motif (Ishibashi et al., 2014). Early crystallographic studies confirmed previous reports that physiologically, aquaporins adopt a tetrameric structure (Mitra et al., 1994; Daniels et al., 1999). Recent native mass spectrometry results have confirmed this tetrameric structure of AQP0 in solution (Harvey et al., 2022).

The water permeability rate varies between aquaporin isoforms; for instance, AQP0 water permeability is approximately 20-fold lower than AQP1 and AQP5 (Chandy et al., 1997; Yang and Verkman, 1997). In addition to transporting water, some aquaporins can transport other molecules such as glycerol (AQPs 3, 7, 9, and 10), ammonia (AQPs 3, 7, 8 and 9), urea (AQPs 7, 9 and possibly 3), and hydrogen peroxide (AQPs 0, 1, 3, 5, 8 and 9) (Bienert et al., 2008; Litman et al., 2009; Ishibashi et al., 2014; Varadaraj and Kumari, 2020). Aquaporins may play other roles in addition to their roles as membrane channels. For example, AQP0 has also been shown to possess cell adhesive properties that are critical to establishing lens transparency and that may be involved in the development the refractive index gradient, that is a key aspect of the refractive properties of the lens (Kumari and Varadaraj, 2009).

## Aquaporins in the Lens

**FIGURE 4:**
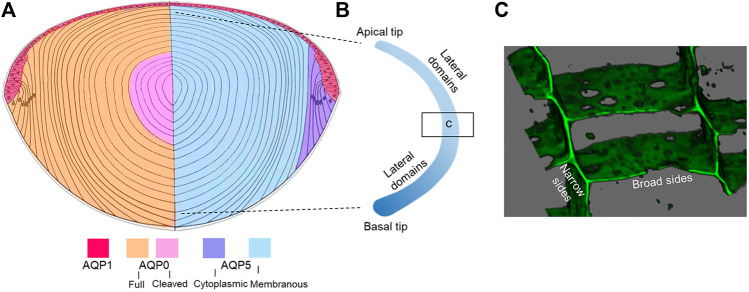
Distribution of lens AQPs. **(A)** Axial cross section showing how the spatial differences in the distribution of AQP1 in the anterior epithelium, full length and cleaved AQP0 (left hemisphere) and cytoplasmic and membraneous AQP5 (right hemisphere). Note that AQP7 and APQ8 are expressed in lens epithelium and are not depicted. **(B)** Schematic of an isolated fiber cell depicting the apical and basal tips that form the anterior and posterior sutures, respectively, and the greatly elongated lateral membranes. Along a fiber cell the distributions of AQP0 and AQP5 in these distinct membrane domains varies as a function of fiber cell differentiation. **(C)** 3D volume rendered image taken from an equatorial section through the lens that has been labelled with the membrane marker WGA showing the narrow and broad side membrane domains where AQP0 and AQP5 are differentially localized.

Five aquaporins have been reported in the lens (AQP0, AQP1, AQP5, AQP7, AQP8) with each protein displaying unique localization and abundance patterns, discussed in detail below (Figure 4A). Since the rate of movement of water across cell membranes, given by the membrane permeability to water (P_H2O_), is enhanced by the presence of AQPs in cell membranes, we can expect that differences in AQP expression, subcellular distribution, lipid and protein interactions, and function will all contribute to the directed movement of water into and out of the lens. In addition, other AQP properties, such as cell adhesion, could also be important in establishing the lens MCS and will be discussed below. There are few reports on lenticular AQP7 and AQP8 expression and both appear to be expressed exclusively in lens epithelial cells (Tran et al., 2013; Hayashi et al., 2017; Varadaraj and Kumari, 2020). Although AQP8 is a known peroxiporin (Bienert et al., 2008), the specific function of AQP7 and AQP8 in the lens has yet to be determined. Thus, our discussion will focus on AQPs 0, 1, and 5.

### Epithelial Cell Aquaporin-1

AQP1 expression is specific to lens epithelial cells and lenses of AQP1-null mice showed opacification and a change in water content demonstrating the role of AQP1 in lens transparency (Ruiz-Ederra and Verkman, 2006). Deletion of AQP1 in the lens epithelium resulted in a threefold reduction of the epithelial water permeability of AQP1 knockout mice lenses (Ruiz-Ederra and Verkman, 2006). The same study reported acceleration of lens opacities in AQP1 knockout lenses organ cultured *in vitro* in high glucose. Recently, it has been reported (Lo et al., 2020) that AQP1 expression in two distinct epithelial regions changes as a function of lens development and growth in mice. In younger lenses (P3-P9) AQP1 expression was located in the central lens epithelium. In contrast, in older lenses, AQP1 expression was increased in the equatorial epithelium and remained in a small area of the central epithelium thereby corresponding to the two major sites for water influx and efflux. Thus, it appears that lens epithelial AQP1 is required to promote water influx and efflux across the epithelium, a function that is necessary to maintain lens transparency, especially following exposure to stress conditions such as hyperglycemia and osmotic imbalance.

### Fiber Cell Aquaporin-0

AQP0 is the most abundant integral membrane protein in the lens making up roughly 50% of the lens membrane proteome (Fitzgerald et al., 1983). AQP0 plays critically important roles in maintaining lens transparency as evidenced by knockout of AQP0 in mouse and mutations of AQP0 in humans leading to congenital cataracts (Berry et al., 2000; Francis et al., 2000; Al-Ghoul et al., 2003; Zeng et al., 2013; Yu et al., 2014), with many of these AQP0 mutations resulting in cataract formation through the development of defects in plasma membrane trafficking (Shiels and Bassnett, 1996; Varadaraj et al., 2008). AQP0 is distributed in both the cortical and nuclear fiber cells where it performs specific regional functions. In the lens cortex, it functions primarily as a water channel and plays a critical role in sustaining the lens microcirculation system. Deeper in the lens core, AQP0’s main function shifts to one of junction formation and cell adhesion (Gonen et al., 2004; Kumari and Varadaraj, 2009). These cell adhesion properties of AQP0 are a result of both AQP0-AQP0 interactions (Gonen et al., 2004) and AQP0-apposing cell membrane (lipid) interactions (Costello et al., 1989; Zampighi et al., 1989; Michea et al., 1994; Kumari et al., 2013), and likely play a role in suture formation (Al-Ghoul et al., 2003). Given that AQP0 has been demonstrated to possess multiple functions including acting as a rather poor water channel (Varadaraj et al., 1999), an adhesion molecule (Kumari and Varadaraj, 2009), and a structural protein linking the plasma membrane to the cytoskeleton (Lindsey Rose et al., 2006; Nakazawa et al., 2011; Wang and Schey, 2011), it is not surprising that the loss of functional AQP0 produces deleterious effects on lens development, suture formation (Al-Ghoul et al., 2003) and overall lens homeostasis. Consistent with this view the replacement of AQP0 with another aquaporin water channel (AQP1) without these additional adhesive and structural functions does not fully rescue the cataract phenotype (Varadaraj et al., 2010; Clemens et al., 2013).

### Epithelial and Fiber Cell Aquaporin-5

A RT-PCR study reported that an AQP5 transcript was expressed at low levels (compared to other lens aquaporin isoforms) in the rat lens (Patil et al., 1997). Subsequent proteomic studies by Wang et al. (2008), and Bassnett et al. (2009), confirmed AQP5 protein expression in the lens. Proteomic studies also showed that AQP5 distribution spans the epithelial, cortical and fiber cell regions of the lens and that the subcellular localization pattern of AQP5 varies depending on the lens region (Wang et al., 2008; Bassnett et al., 2009; Grey et al., 2013). Specifically, AQP5, in contrast to AQP0, does not immediately insert into the membranes of differentiating lens fiber cells (Petrova et al., 2015). Importantly, lens vesicles show increased water permeability when AQP5 is present in their membranes (Petrova et al., 2018). This result suggests that water permeability changes from the outer cortex to the inner cortex. Further, when combined with evidence of AQP5 trafficking in response to zonular tension (Petrova et al., 2020), these results suggest that AQP5 can dynamically regulate lens fiber cell water permeability, at least in the outer cortex of the lens. Recently, AQP5, as well as AQP0 and AQP1, have been shown to function as peroxiporins that are permeable to hydrogen peroxide (Varadaraj and Kumari, 2020). The fact that AQP5 knockout animals are cataractous (Tang et al., 2021), and are susceptible to osmotic stress-induced cataract (Sindhu Kumari and Varadaraj, 2013), suggests that control of AQP5 permeability is important in regulating fiber cell volume and water homeostasis.

## Multiple Mechanisms Regulate Lens Aquaporin Function

As fiber cells differentiate from lens epithelial cells at the lens equator, we expect normal differentiation-related processes, such as protein synthesis/degradation, protein trafficking and post-translational modification, to govern protein expression and function. However, fully mature lens fiber cells are organelle-free and therefore lack the ability to synthesize new protein (Bassnett and Costello, 2017). Thus, regulation of protein function in mature fiber cells is accomplished by post-translational modifications, protein-protein interactions, or by changes in the local environment (Schmid and Hugel, 2020). Layered on top of these regulatory processes is a set of additional changes that occur to lens AQPs as we age. Because the lens continues to grow throughout life, an age gradient is established where the oldest cells are found in the lens core and the youngest cells are found in the outer cortex. Accumulation of age-related lens protein modifications have been studied for multiple decades (Takemoto and Takehana, 1986; Garland et al., 1996; Lampi et al., 1998; Ueda et al., 2002; Schey et al., 2020). Interestingly, modifications, as well as protein interactions and lipid environment, change throughout the lens suggesting that the functionality and regulation of lens AQPs also changes as a function of their location (i.e., age) in the lens. Such age-related changes could lead to changes in the local properties of AQPs that may contribute to the development of age-related cataract. In the next two sections we first highlight mechanisms known to regulate lens AQP functionality before discussing how age-related changes can alter this regulation in different areas of the lens.

### Mechanisms That Regulate Lens Aquaporin Function

The following mechanisms have been shown to alter AQP function and their potential roles in lens AQP regulation are discussed:

*Phosphorylation:* Reversible protein phosphorylation is one of the most common posttranslational modifications and provides dynamic posttranslational control of protein function (Hardie, 1989). In lens fiber cells, both AQP0 and AQP5 are phosphorylated (Ball et al., 2004; Kumari et al., 2012; Schey et al., 2000; Wang et al., 2013), hence phosphorylation may play important roles in regulating AQP water permeability, membrane trafficking, and/or cell-cell adhesion. Three AQP0 phosphorylation sites (S229, S231, and S235) were identified in the short amphipathic helix (Leu227-Gly237) located in the AQP0 C-terminus (Ball et al., 2004; Schey et al., 2000) (Figure 3). S235 is the major phosphorylation site and phosphorylation at S229 and S231 are present at significantly lower stoichiometries (Gutierrez et al., 2011). AQP0 P_H2O_ is known to be reduced by calmodulin (CaM) binding and the interaction of CaM through binding to the short amphiphilic helix region (Leu227-Gly237) of AQP0 is reduced upon phosphorylation (Girsch and Peracchia, 1991; Rose et al., 2008). Phosphorylation, especially at S235, severely impaired CaM-AQP0 interaction (Reichow and Gonen, 2008; Rose et al., 2008), therefore, phosphorylation of AQP0 to impede CaM binding would be expected to increase AQP0 P_H2O_. Fields et al. (2017) reported a second site of contact between AQP0 and CaM in the arginine-rich intracellular loop (ICL2, Figure 3) where CaM allosterically controls the dynamics and configuration of the pore opening. Thus, phosphorylation not only regulates AQP0 permeability by modulating CaM-AQP0 interactions but also through allosteric mechanisms that directly alter AQP0 P_H2O_. Quantification of AQP0 phosphorylation in the different regions of the lens showed that phosphorylation, especially phosphorylation on the major phosphorylation site S235, is spatially regulated (Ball et al., 2004; Gutierrez et al., 2016). Specifically, phosphorylation levels on S235 are low in the outer cortex and peak in the inner cortex region before decreasing in the lens core (Ball et al., 2004; Gutierrez et al., 2016). Therefore, AQP0 S235 phosphorylation could play an important role for establishing the lens microcirculation system by restricting P_H2O_ in the outer cortex and by increasing permeability in the inner cortex through to the lens core.

In AQP5, two consensus PKA sites are found: S156 in ICL2 in Figure 3 (aa 153–157) and T259 in the C-terminus. While phosphorylation of T259 has been confirmed in lens fiber cells, a quantitative assessment of AQP5 phosphorylation through the lens has not been done. Phosphorylation of S156 has not been detected in the lens despite phosphopeptide enrichment and global phosphoproteomic analysis (Wang et al., 2013). In addition to regulating the inherent P_H2O_ of AQP water channels, phosphorylation has also been shown to dynamically modulate AQP trafficking (van Balkom et al., 2002; Nesverova and Tornroth-Horsefield, 2019) and alter the abundance of water channels in the membrane and therefore P_H2O_ (discussed below). The classic example is the phosphorylation of the AQP2 C-terminal residue S256 that promotes the trafficking of AQP2 to the apical membrane of epithelial cells that line the cortical collecting duct of the kidney (van Balkom et al., 2002). In this regard it is interesting to note that T259 in AQP5 is a site that is considered homologous to S256 in AQP2. In non-lens cells, AQP5 trafficking to the plasma membrane has been shown to be both dependent (Yang et al., 2003; Kumari et al., 2012) and independent (Hasegawa et al., 2011) of phosphorylation status. In short, the phosphorylation status of lens cytoplasmic and membrane AQP5 remains an unanswered question.

*Membrane Trafficking:* As mentioned above, phosphorylation-dependent trafficking to and from the membrane may be a way to modulate P_H2O_ of fiber cell membranes, however it needs to be remembered that fiber cells are essentially elongated epithelial cells that retain their distinct apical and basal membrane domains but have dramatically elongated lateral membranes (Figure 4B). To achieve an orderly packing that minimizes extracellular space, fiber cells adopt a hexagonal cross-sectional shape and lateral membranes are further subdivided into distinct broad and narrow side membrane domains (Figure 4C) (Zampighi et al., 2000). The apical domains of elongating fiber cells meet at the anterior pole and the basal domains at the posterior pole (Kuszak et al., 2004) to form the lens sutures, which create extracellular pathways linking the central core of the lens to the surrounding humours that supply the lens with nutrients. Hence the trafficking of AQP0 and AQP5 to these distinct membrane domains appears to be differentially regulated. Immunofluorescence studies revealed that AQP0 phosphorylation (S235) was needed for trafficking to the plasma membrane and that based on PKC inhibitor studies, PKC was associated with its trafficking (Golestaneh et al., 2008). However, AQP0 appears to immediately traffic to the plasma membrane upon synthesis in the earliest differentiating fiber cells. Interestingly, proper trafficking and localization of AQP0 may be dependent on Eph-Ephrin signaling (Cheng et al., 2021).

In contrast, AQP5, a close homologue of AQP2, appears to traffic by a different mechanism from that of AQP0 (Gletten et al., 2022). AQP5 displays a cell-dependent localization pattern where it is predominantly found in the cytoplasm of differentiating fiber cells and in the plasma membrane of mature fiber cells (Grey et al., 2013; Petrova et al., 2015; Gletten et al., 2022). Within individual fiber cells of the same lens region, differences in the trafficking of AQP5 to the apical and basal membrane tips of fiber cells, corresponding to the anterior and posterior sutures, was observed (Petrova et al., 2020). In contrast to AQP0, AQP5 displays a clear change in subcellular localization upon alteration of zonular tension suggesting it may help the lens adapt under conditions of stress (Petrova et al., 2020). Pharmacological interventions with TRP channel activators/inhibitors suggest that mechano-sensing TRP channels can regulate AQP5 localization in the equatorial efflux and anterior influx zones of the rat lens (Petrova et al., 2020). Importantly, fiber cell membrane permeability correlates with the amount of plasma membrane AQP5 present (Petrova et al., 2018). Whether these dynamic changes in AQP5 membrane localization are associated with changes in the phosphorylation of AQP5, as has been determined for its close homolog AQP2 in the collecting ducts of the kidney (Jung and Kwon, 2016; Moeller et al., 2016), remains to be determined.

*Protein Interactions:* Multiple proteins have been reported to interact with AQP0 and such interactions occur most often through the AQP0 C-terminus (Figure 3). As mentioned above, AQP0 water permeability is regulated by interaction with CaM through the amphiphilic helix (Girsch and Peracchia, 1991; Lindsey Rose et al., 2006) and positively charged arginine-rich loop (Fields et al., 2017). In addition, proteins that interact with AQP0, for example, filensin, also undergo modifications and change subcellular localization. Therefore, protein-protein interactions are expected to be highly spatially controlled in lens fiber cells, and thus it is important to consider where in the lens these interactions occur as well as their potential effects on P_H2O_ in those specific regions of the lens.

AQP0 associates with gap junction plaques in a narrow zone of the lens bow region and this interaction may facilitate the assembly of Cx50 into nascent gap junction plaques during the early stages of embryonic lens development (Yu and Jiang, 2004). The AQP0-Cx50 interaction enhances the formation of functional gap junction channels (Liu et al., 2011) predicted to be important in establishing the outflow pathway of the MCS. This interaction occurs via the C-terminus of AQP0 and the intracellular loop region of Cx50 (Liu et al., 2011) and is abolished with age-related truncation of both proteins (Wenke et al., 2015; Slavi et al., 2016). Multiple cytoskeletal proteins have been reported to interact with AQP0 through its C-terminal tail including filensin (Wang and Schey, 2017), CP49 (Lindsey Rose et al., 2006) and ezrin (Wang and Schey, 2011). Importantly, the AQP0-filensin interaction can affect AQP0 water permeability (Nakazawa et al., 2011). The AQP0-filensin interaction is another example of spatially regulated protein-protein interactions in the lens since regional (age-related) truncation of filensin occurs (Sandilands et al., 1995; Wang et al., 2010). Spatially resolved proteomic analysis indicated ezrin and radixin are among the very few proteins that increase membrane association together with increasing AQP0 expression and formation of extracellular diffusion barrier in the inner cortex of the bovine lenses (Wang et al., 2021). Ezrin is one of the components of the ezrin, periplakin, periaxin, desmoyokin (EPPD) adherens complex (Straub et al., 2003). Together with increasing ezrin membrane association, periplakin and desmoyokin abundances decreased significantly. It is reasonable to predict that ezrin is released with the decomposition of EPPD complexes and reused by the fiber cells to regulate AQP0 membrane redistribution (Wang et al., 2021).

Studies of proteins that interact with AQP5 in the lens are limited, however, in other tissues, AQP5 function can be regulated by protein-protein interactions (Ohashi et al., 2008; Chivasso et al., 2021a). For example, the AQP5 C-terminus was found to interact with prolactin-inducible protein (PIP) in control mice (Jcl:ICR, CLEA Japan), but interact with major urinary protein 4 in non-obese diabetic (NOD) mice (model for Sjögren’s syndrome) (Ohashi et al., 2008). The PIP-AQP5 interaction plays an important role in controlling AQP5 localization in human salivary glands (Chivasso et al., 2021b). AQP5/ezrin interaction in salivary glands was also reported and this interaction could be involved in the regulation of AQP5 trafficking and may contribute to AQP5-altered localization in Sjögren’s syndrome patients (Chivasso et al., 2021b). Since ezrin is abundant in lens fiber cells, it will be interesting to study if AQP5 also interacts with ezrin and where in the lens such an interaction occurs.

*Lipid Interactions:* Intrinsic membrane proteins are embedded in a biological membrane where lipids dynamically interact with them and can affect their function as well as structural properties such as folding, packing and stability (Lee, 2004). Similar to other cell types, we have shown that lens fiber cell proteins exist in lipid rafts or non-raft environments (Wang and Schey, 2015). Others have demonstrated that different lipids can affect AQP stability (Laganowsky et al., 2014) and permeability (Tong et al., 2013). Although lipid changes with fiber cell age have been reported (Borchman and Yappert, 2010; Hughes et al., 2012), how different lens lipids interact with lens aquaporins in specific lens regions to regulate their function in the context of the lens microcirculatory system requires further exploration.

### Effects of Age-Dependent Modifications on Lens Aquaporin Function

Numerous studies of age-related modifications to lens proteins reveal extensive age-related modifications to AQPs including truncation (Takemoto and Takehana, 1986; Schey et al., 2000; Ball et al., 2004; Gutierrez et al., 2011), deamidation (Ball et al., 2004; Wenke et al., 2015), and crosslinking (Friedrich et al., 2019; Wang et al., 2019). How these age-dependent modifications affect AQP regulation and function in the different regions of lens and therefore overall lens function remains unknown. The following age-related modifications have been identified.

*Truncation:* Non-enzymatic truncation is a prevalent age-related modification observed for most of the abundantly expressed lens proteins, including AQP0 (Schey et al., 2000) and AQP5 (Schey, unpublished results). Given the importance of the AQP C-terminal tail in regulating permeability and in AQP-protein interactions, loss of this portion of the protein through the aging process is expected to have a significant impact on both permeability and the ability to regulate water movement.

AQP0 undergoes extensive C-terminal truncation with two major truncation sites at Asn 246 and Asn 259 (Takemoto and Takehana, 1986; Schey et al., 2000; Ball et al., 2004; Gutierrez et al., 2011). Truncation increases steadily with fiber cell age from the lens cortex to the lens nucleus, and plateaus in regions of lens nucleus (Gutierrez et al., 2011; Wenke et al., 2015) such that 50% of AQP0 is truncated at a fiber cell age of around 25 years (Gutierrez et al., 2011). Considering the extensive truncation of AQP0 observed in older, but still transparent lenses, truncation is most likely a normal age-related event and not necessarily cataractogenic. However, when the rates and sites of truncation change, AQP0 truncation could be detrimental to the lens function. For example, accelerated AQP0 C-terminal truncation can be detected in hyperbaric oxygen treated guinea pig lenses, a model for nuclear cataract development (Giblin et al., 2021). Cleavage of the AQP0 C-terminus has been reported to enhance the adhesive properties of the extracellular surface of AQP0 and promote the formation of AQP0-AQP0 junctions (Gonen et al., 2004). The cleaved form of AQP0 was predicted to have a lower water permeability than intact AQP0; however, when truncated AQP0 proteins were expressed in oocytes (Ball et al., 2004; Kumari and Varadaraj, 2014 #80), no difference in water permeability or cell-cell adhesion was found compared to full length AQP0. This finding led Kumari and Varadaraj (2014) to predict that AQP0 truncation may play a role in adjusting the refractive index to prevent spherical aberration in the constantly growing lens. Since the AQP0 C-terminus is the region of AQP0 that interacts with multiple cytoskeletal proteins (Lindsey Rose et al., 2006; Wang and Schey, 2011; Wang and Schey, 2017), C-terminal truncation is expected to impair such interactions. As suggested above, AQP0 and cytoskeletal protein interaction could be localized in very specific regions of the lens and truncation of AQP0 could then play a regulatory role for AQP0 C-terminal involved protein-protein interactions.

Consistent with western blot results, AQP5 undergoes only minor C-terminal truncation in the lens core (Grey et al., 2013; Petrova et al., 2015). Using highly sensitive mass spectrometry techniques, some truncation of AQP5 at residues F199, T264, D248, D246 and E244 in the human lens core can be detected (Schey, unpublished data). The functional consequences of AQP5 truncation have not been studied, but loss of the major AQP5 phosphorylation site (T259) is expected to have functional consequences in regard to protein interactions.

*Deamidation:* One of the most abundant age-related modifications in human lenses is deamidation (Kim et al., 2002; Lampi et al., 2006; Takata et al., 2008) and AQP0 is known to undergo extensive deamidation (Schey et al., 2000; Ball et al., 2004) even in lenses as young as 4 months old (Wenke et al., 2015). The functional consequences of AQP0 C-terminal deamidation remain to be determined, however, considering deamidation occurs in the region where AQP0 interacts with several cytoskeletal proteins (Lindsey Rose et al., 2006; Wang and Schey, 2011; Wang and Schey, 2017), this modification could alter AQP0 function in different regions of the lens. Furthermore, most AQP0 deamidation occurs on the C-terminus; however, deamidation was also detected on Asn115 in the second extracellular loop, Gln129 in a transmembrane domain and Asn197 and Asn200 in the third extracellular loop (Schey, unpublished data). The extracellular loops of AQP0 are important in regulating AQP0 cell adhesion function (Gonen et al., 2004; Kumari et al., 2019); therefore, extracellular loop deamidation could affect AQP0 cell adhesion function.

*Crosslinking:* It has been widely recognized that lens protein crosslinking and loss of solubility contribute to the development of age-related lens opacity (Dilley and Pirie, 1974). Recently, with the development of high-resolution mass spectrometry as well as improvements in crosslinked peptide searching algorithms, direct analysis of residues involved in crosslinking has become possible. Five distinct crosslinking mechanisms have been elucidated in aged lens fiber cells (Wang et al., 2014; Friedrich et al., 2018; Friedrich et al., 2019; Wang et al., 2019) and AQP0, given its high abundance, is among the proteins that are frequently crosslinked. The regions of AQP0 involved in crosslinking include the N-terminal amino group, Lys228 and C-terminal Asn or Asp residues. Some AQP0 crosslinks can be detected in the lens nucleus in lenses as young as 20 years old (Friedrich et al., 2019; Wang et al., 2019). AQP0 is frequently detected to be crosslinked with itself, presumably forming crosslinks within a single AQP0 tetramer. In addition, AQP0 crosslinked with γS crystallin has been detected (Wang et al., 2019). The functional consequence of AQP0 crosslinking has not been studied; however, Lys228 is within the amphiphilic helix where CaM binds with AQP0 (Girsch and Peracchia, 1991; Rose et al., 2008; Fields et al., 2017). Thus, crosslinking of AQP0 is expected to change interaction with CaM and may directly affect AQP0 permeability.

## Regional Changes in AQUAPORIN Structure and Function Contribute to Lens Water Transport: An Updated Model

Experimental confirmation of the existence of water fluxes (Mathias et al., 1997; Mathias et al., 2007; Donaldson et al., 2010; Gao et al., 2011; Candia et al., 2012; Vaghefi et al., 2012) along with evidence of their dynamic regulation (Gao et al., 2015) led to the development of an initial model of how regional differences in lens aquaporin expression, localization, and regulation combine to produce regional differences in fiber cell P_H2O_ that facilitate the outflow and efflux of water from the lens driven by the local osmotic gradients generated by the ion fluxes that drive the MCS (Schey et al., 2017). However, this model was based solely on regional differences in lens AQP functionality and did not consider that fiber cells within a specific lens region also exhibit differences in the distribution of AQP0 and AQP5 that occur in the different membrane domains observed along the length of individual fiber cells (Figure 4B) (Zampighi et al., 2002; Grey et al., 2009; Petrova et al., 2020). Thus, AQP channels in the anterior and posterior tips of fiber cells located at the anterior and posterior poles of the lens will mediate water influx, while in the same cell, water flow across the lateral membranes will be directed out of the lens *via* an intracellular pathway mediated by gap junctions where at the lens surface AQP channels mediate the efflux of water from the lens. In this section, we now present an updated model of regional differences in AQP functionality which includes recently acquired data (Petrova et al., 2020) that suggest differences in localization and regulation of AQP5 in the anterior and posterior sutures differentially modulates the influx of water at the anterior and posterior poles. While this model attempts to show how regional differences in the molecular and cellular structure and function of lens AQPs contributes to overall function of the lens at the whole tissue level, like all models it is at best an approximation of the real situation and will require further experimental validation.

**FIGURE 5:**
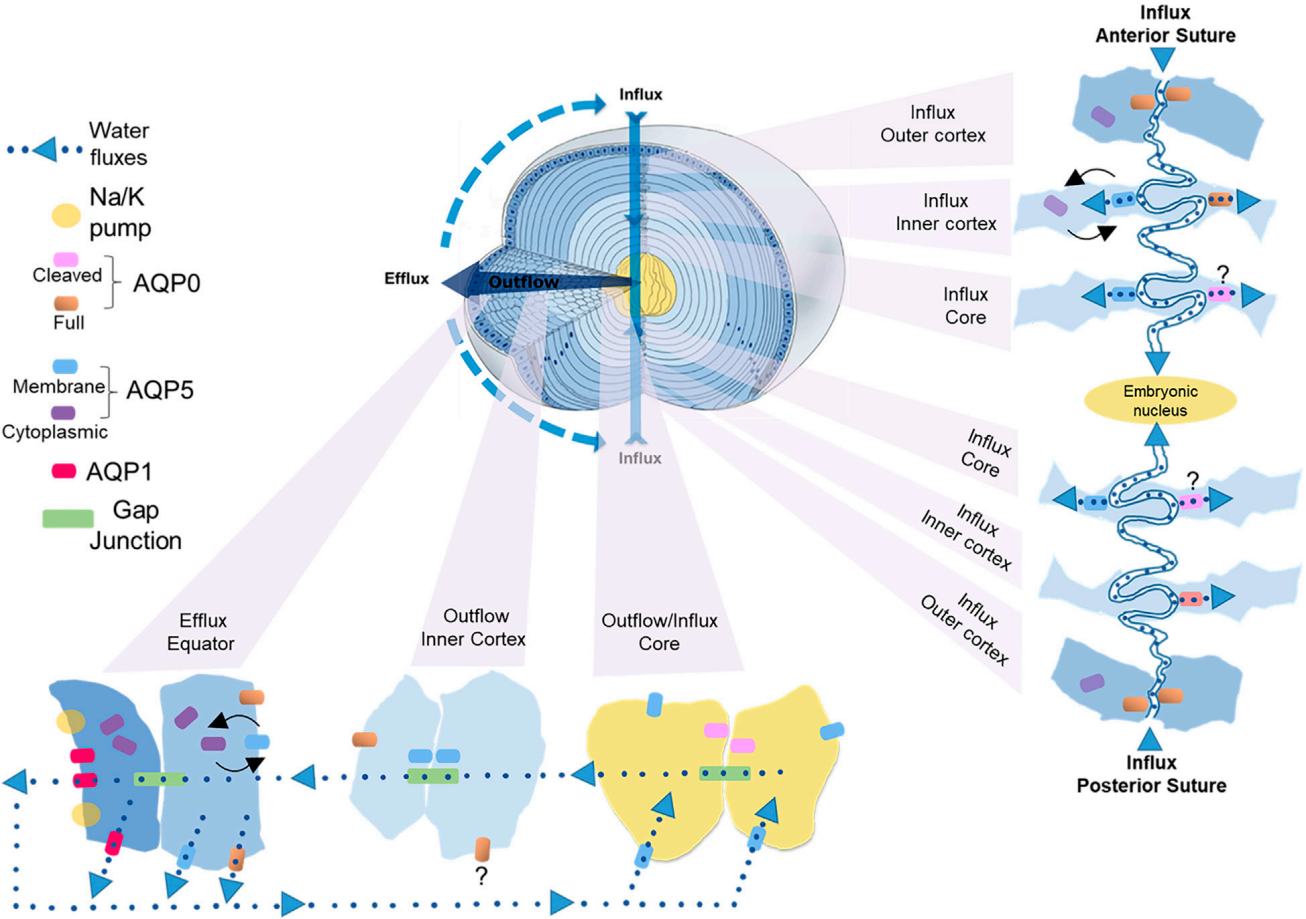
Cellular and regional differences in AQP expression that contribute to the water influx, outflow and efflux in the lens. Apical and basal tips from adjacent fiber cells interact to form the anterior and posterior sutures, respectively, that function as an extracellular influx pathway that directs ions and water into the lens and traverses the lens extracellular diffusion barrier to deliver water to the lens core. The fiber cell tips of both sutures contain AQP0, but only the apical tips of fiber cells in inner cortex that form the anterior suture contain AQP5. In the inner cortex of this anterior influx zone changes in zonular tension dynamically regulate the trafficking of AQP5 to the apical tips of fiber cells to modulate the flow of water from the anterior suture into fiber cells in this region. In the lens core we propose that water uptake is mediated mainly by AQP5 rather than truncated AQP0. Once delivered to the lens core water flows out towards the equatorial surface for the lens *via* an intracellular pathway mediated by gap junctions. In the inner cortical region of this outflow pathway we propose that the P_H2O_ of fiber cells is reduced relative to the P_H2O_ of gap junctions to facilitate the cell-to-cell movement of water. This reduction in plasma membrane PH2O is facilitated in part by recruitment of AQP0 to junctional structures that restrict the extracellular space and AQP5 to plaque like structures on the broad sides of fiber cells that have a similar distribution to gap junction plaques. Once water reaches the lens periphery it can efflux the lens via AQP1 and AQP5 in epithelial cells and AQP5 and AQP0 in differentiating fiber cells located at the lens equator. In this efflux zone P_H2O_ can also be dynamically regulated by changes in AQP5 membrane trafficking in response to changes in zonular tension.

In this updated model (Figure 5), AQP1 is a constitutively active water channel exclusively localized to lens epithelial cells, where it mediates water influx and efflux in the central and equatorial regions of the lens, respectively. In contrast, fiber cell P_H2O_ is dependent on both AQP0 and AQP5, which undergo distinctly different differentiation-dependent changes in their subcellular location and post-translational modification which by altering local P_H2O_ contribute to the overall magnitude and directionality of water fluxes that circulate through the lens. Based on our current understanding of these changes to AQP0 and AQP5 expression we propose that full length AQP0 provides a basal level of water permeability in the outer lens cortex and this permeability can be altered by AQP0 phosphorylation/calmodulin binding (Lindsey Rose et al., 2006; Fields et al., 2017). In this outer region of the lens, AQP5 appears to operate as a regulated water channel, where its membrane location, and hence contribution to fiber cell P_H2O_, can be modulated by either mechanically or pharmacologically altering zonular tension *via* a process that is mediated by TRP channels (Petrova et al., 2018; Petrova et al., 2020). In the lens inner cortex, AQP0 forms cell-cell junctions, either AQP0-AQP0 junctions or AQP0-plasma membrane junctions (Zampighi et al., 1989; Gonen et al., 2004), that may enable the formation of an extracellular diffusion barrier (Grey et al., 2009). Recent proteomics analysis of the barrier region indicates that adhesion proteins also play a role in barrier formation (Wang et al., 2021). A major consequence of this barrier formation is that delivery of water and nutrients to the lens core occurs via the lens sutures (Vaghefi and Donaldson, 2018). Further, AQP modifications that reduces the P_H2O_ of the plasma membrane relative to the P_H2O_ of the gap junctions would tend to facilitate the cell-to-cell removal of water via the intracellular outflow pathway mediated by gap junctions. In this regard it has been shown that AQP0 becomes associated with the periphery of gap junction plaques in this region (Grey et al., 2009). In contrast, we have preliminary data for AQP5 that shows in the inner cortex AQP5 accumulates in plaque-like structures on the broadsides of fiber cells (unpublished data) that resemble the gap junction plaques known to form in this region of the lens (Jacobs et al., 2004). Whether AQP5 also contributes to the intracellular outflow of water from the core that is thought to be mediated by gap junctions (Gao et al., 2011) remains to be determined. In the lens core, extensive age-related modifications such as truncation of the C-terminus of AQP0 will change the regulation of AQP0 P_H2O_ permeability by phosphorylation/calmodulin binding and interaction with binding partners, while the altered lipid environment in the lens is predicted to reduce the P_H2O_ of AQP0 based on *in vitro* studies (Tong et al., 2013). Since it appears that the majority of C-terminus of AQP5 is largely intact in the lens core (Grey et al., 2013; Petrova et al., 2015), we propose that the bulk of water transport occurs *via* full length AQP5 in the core rather than *via* truncated AQP0 water channels.

Since the diffusion of water through water channels is driven by the local osmotic gradient established by ion transport we will now propose how these observed spatial differences in AQP0 and AQP5 location and functionality combine to drive water transport through the lens (Figure 5). In parallel to ion movement, water enters the lens at both poles via an extracellular route that is associated with the sutures (Candia et al., 2012; Vaghefi et al., 2011). This influx zone spans from the lens surface to the core of the lens (Figure 5, influx pathway) and crosses the extracellular diffusion barrier formed in the inner cortex (Vaghefi and Donaldson, 2018; Vaghefi et al., 2012). Since in the outer cortical region of this influx zone AQP5 is not associated with the apical or basal tips of fiber cells (Petrova et al., 2020), we envisage that water will only enter fiber cells *via* AQP0 water channels (Figure 5, anterior influx—outer cortex). In addition, it is possible water will remain in the extracellular space between fiber cells as it moves away from the sutures towards the equatorial regions of the lens before crossing fiber cell membranes *via* AQP0 and/or AQP5 channels located in the lateral membranes of fiber cells. In the inner cortex the restriction of extracellular space would reduce this lateral extracellular movement of water away from the sutural influx zone and direct water flow towards the lens core. In this inner cortical region movement of water from the suture into the fiber cells *via* AQP5 can be dynamically modulated by changes in zonular tension in the anterior (Figure 5, anterior influx–inner cortex), but not the posterior sutural influx zone (Petrova et al., 2020). We speculate that this ability to differentially change water influx in this region of the lens may be a mechanism via which the lens can change the curvature of its anterior surface and hence the optical properties of the lens. In the core of the lens the water delivered to this zone would then be taken up into fiber cells via both AQP0 and AQP5 (Figure 5, influx/outflow—core), however as mentioned above, we are currently unsure about the relative contributions of truncated AQP0 and non-truncated AQP5 to fiber cell P_H2O_ in this region of the lens.

Once delivered to the central lens, water moves towards the surface of the lens *via* an intracellular outflow pathway that delivers water to an equatorial efflux zone where water leaves the lens. In this outflow pathway (Figure 5, outflow—inner cortex) the gradients for water movement are generated by both the movement of ions through gap junctions and the hydrostatic pressure gradient generated by the outflow of water. In this area of the lens we envisage that limiting the flow of water across fiber cell membranes into the extracellular space by altering the functionality of AQP0/AQP5 via post-translational modifications will promote the intracellular passage of water through gap junction channels. Finally, once water reaches the equatorial efflux zone it can leave the lens through AQP1 or AQP5 channels located in equatorial epithelial cells or by AQP0 or AQP5 channels in peripheral fiber cells (Figure 5, outflow—equator). Since the subcellular distribution of AQP5 can be dynamically altered by mechanically or pharmacologically altering zonular tension via a process that is mediated by TRP channels (Petrova et al., 2020), P_H2O_ of fiber cells in the efflux zone can be regulated in parallel to the ion fluxes that drive the transport of water throughout the lens. Taken together, it is clear that the expression level, the subcellular localization, the extent of modification, the local lipid environment, and the extent of protein-protein interactions all play roles in the regulation lens water transport which has been shown to be so critical for the maintenance of the transparent and refractive properties of the lens (Donaldson et al., 2017).

## Lens Water Transport—A New Target for Anti-Cataract Drug Discovery

Based on our knowledge that AQP mutations or AQP deletion leads to cataract formation (Shiels and Bassnett, 1996; Shiels et al., 2000; Okamura et al., 2003; Tang et al., 2021) and that AQPs are an integral component of lens water transport, we propose that age-related modifications to AQP functionality are involved in cataract formation. Consistent with this view, glycemic stress in AQP5 knockout mice leads to cataract, suggesting a protective role of AQP5 under conditions of stress (Sindhu Kumari and Varadaraj, 2013). Thus, if AQP age-related modifications and diabetic stress lead to altered AQP function, then we surmise that age-related opacification is a possible consequence. It is also conceivable that age-related changes in water transport could affect lens stiffness and, as such, AQPs could represent a novel target for presbyopia treatment.

In other tissues where AQP dysfunction has been linked to numerous diseases, AQPs have been promoted as therapeutic drug targets (Verkman et al., 2014; Soveral and Casini, 2017; Salman et al., 2022). For example, the involvement of AQPs in cancer initiation, cell migration and tumor angiogenesis, has made AQPs attractive targets for novel anticancer therapies (Wang et al., 2015; Elkhider et al., 2020). AQP1 inhibitors have also been considered as a therapeutic target for the treatment of intraocular hypertension in glaucoma (Patil et al., 2018). Thus, therapeutic targeting of aquaporins is an active area of research (Villandre et al., 2022), including in ocular tissues, and we can expect continued discoveries of new AQP modulators. In the context of lens water transport and cataractogenesis, the lens microcirculation system needs to be tightly regulated and it is worth considering lens AQPs as a potential anti-cataract targets (Pierscionek, 2021). In the search for natural products for cataract treatment, *Heliotropium indicum* extract was found to alleviate selenite-induced cataract and also increase AQP0 levels (Kyei et al., 2015). Considering the increased water content in the aged lens nucleus (Siebinga et al., 1991), the accumulation of water and sodium, and the swelling of fiber cells in AQP5 deficient lenses under hyperglycemic stress (Sindhu Kumari and Varadaraj, 2013), therapeutics designed to increase efflux of water could be used to alleviate cataractous conditions.

## Questions and Future Directions

There are many features of our working model for lens water transport that require further investigation. Given the known effects of post-translational modifications, lipid environment and expected effects on local water permeability, spatial definition of these lens properties is necessary to define the molecular basis of lens water transport. It is important to recognize that many previous studies have used either homogenized whole lenses or separation methods with low spatial resolution (Ball et al., 2004; Gutierrez et al., 2016) resulting in limited information on spatial regulation of lens water transport. Furthermore, most spatially-resolved lens studies have examined equatorial sections (Wenke et al., 2015; Wang et al., 2021); however, there may be axial protein differences where expression or modifications occur along the length of individual fiber cells (Zampighi et al., 2002; Kuszak et al., 2004). Thus, spatially-resolved methods of analysis are necessary to increase our understanding of AQP structure and function in the different regions of the lens. In addition, as described above, AQP structure and function can be affected by age. We predict, therefore, that the combined local effects of protein modification, protein-protein interactions, lipid environment, and age-related alterations will affect global lens water transport; a property that has yet to be measured as a function of age. With a better understanding of AQP function as a function of age, we expect that new directions in the treatment of presbyopia and cataract will emerge.
